# Supplementary material for: Effects of sequential feeding with adjustments to dietary amino acid concentration according to the circadian rhythm on the performance, body composition, and nutrient balance of growing-finishing pigs
Source: PLoS One. 2021 Dec 23;16(12):e0261314. doi: 10.1371/journal.pone.0261314 (PMC8700050; doi:10.1371/journal.pone.0261314)
Supplement: S1 Table — (DOCX) [file pone.0261314.s001.docx]

**S1 Table**. **Analyzed nutritional composition of soybean meal and corn.**

|  | **Nutritional composition, %** | |
| --- | --- | --- |
|  | **Soybean meal** | **Corn** |
| Dry matter^1^ | 89.68 | 89.21 |
| Crude protein^1^ | 46.55 | 9.39 |
| Isoleucine^1^ | 2.14 | 0.32 |
| Leucine^1^ | 3.55 | 1.19 |
| Lysine^1^ | 2.84 | 0.27 |
| Methionine^1^ | 0.60 | 0.19 |
| Methionine + cystine^1^ | 1.27 | 0.39 |
| Threonine^1^ | 1.80 | 0.33 |
| Tryptophan^1^ | 0.62 | 0.06 |
| Valine^1^ | 2.22 | 0.45 |

^1^ Near-infrared spectroscopy (NIRS)
