# Supplementary material for: Effects of sequential feeding with adjustments to dietary amino acid concentration according to the circadian rhythm on the performance, body composition, and nutrient balance of growing-finishing pigs
Source: PLoS One. 2021 Dec 23;16(12):e0261314. doi: 10.1371/journal.pone.0261314 (PMC8700050; doi:10.1371/journal.pone.0261314)
Supplement: S2 Table — (DOCX) [file pone.0261314.s002.docx]

**S2 Table. Performance of the experimental pigs.**

| **Variables^1^** | **Mean** | **Minimum** | **Maximum** | **Standard deviation** | **Coefficient of variation (%)** |
| --- | --- | --- | --- | --- | --- |
| **Initial Conditions** |  |  |  |  |  |
| BW, kg | 25.327 | 17.600 | 32.400 | 3.033 | 11.978 |
| **Phase 1 (25–50 kg BW)** |  |  |  |  |  |
| ADFI, kg/day | 1.431 | 0.742 | 2.085 | 0.297 | 20.799 |
| AFI P1, kg/period | 0.648 | 0.289 | 0.991 | 0.172 | 26.507 |
| AFI P2, kg/period | 0.783 | 0.315 | 1.349 | 0.195 | 25.021 |
| SID Lys intake, g/day | 13.672 | 6.733 | 20.356 | 2.898 | 21.199 |
| ADG, kg/day | 0.707 | 0.342 | 1.103 | 0.157 | 22.184 |
| G:F, kg/kg | 0.495 | 0.402 | 0.797 | 0.058 | 11.722 |
| BW, kg | 45.165 | 28.600 | 59.100 | 6.276 | 13.896 |
| **Phase 2 (50–70 kg BW)** |  |  |  |  |  |
| ADFI, kg/day | 2.086 | 1.001 | 2.950 | 0.464 | 22.265 |
| AFI P1, kg/period | 1.006 | 0.438 | 1.670 | 0.269 | 26.807 |
| AFI P2, kg/period | 1.080 | 0.408 | 1.947 | 0.344 | 31.904 |
| SID Lys intake, g/day | 17.013 | 7.842 | 24.591 | 3.917 | 23.027 |
| ADG, kg/day | 0.836 | 0.261 | 1.246 | 0.218 | 26.171 |
| G:F, kg/kg | 0.397 | 0.261 | 0.455 | 0.038 | 9.754 |
| BW, kg | 66.657 | 35.800 | 91.500 | 11.395 | 17.096 |
| **Phase 3 (70–100 kg BW)** |  |  |  |  |  |
| ADFI, kg/day | 2.568 | 1.363 | 3.398 | 0.441 | 17.193 |
| AFI P1, kg/period | 1.198 | 0.660 | 1.951 | 0.283 | 23.652 |
| AFI P2, kg/period | 1.370 | 0.344 | 2.201 | 0.365 | 26.645 |
| SID Lys intake, g/day | 18.289 | 9.535 | 24.212 | 3.267 | 17.865 |
| ADG, kg/day | 1.005 | 0.489 | 1.321 | 0.031 | 17.714 |
| G:F, kg/kg | 0.392 | 0.308 | 0.540 | 0.036 | 9.364 |
| BW, kg | 94.803 | 49.500 | 127.70 | 15.146 | 15.976 |
| **Global performance (25–100 kg BW)** |  |  |  |  |  |
| ADFI, kg/day | 2.024 | 1.046 | 2.728 | 0.376 | 18.586 |
| AFI P1, kg/period | 0.949 | 0.492 | 1.440 | 0.208 | 21.963 |
| AFI P2, kg/period | 1.075 | 0.487 | 1.801 | 0.276 | 25.718 |
| Daily SID Lys intake, g/day | 16.267 | 8.141 | 22.116 | 3.149 | 19.362 |
| ADG, kg/day | 0.848 | 0.370 | 1.213 | 0.164 | 19.442 |
| G:F, kg/kg | 0.418 | 0.354 | 0.476 | 0.027 | 6.521 |

^1^ BW, body weight; ADFI, average daily feed intake; AFI P1, average feed intake in period 1 from 00:00 to 11:59 h; AFI P2, average feed intake in period 2 from 12:00 to 23:59 h; SID, standardized ileal digestible; Lys, lysine; ADG, average daily gain; G:F, gain:feed ratio.
