# Supplementary material for: Effects of sequential feeding with adjustments to dietary amino acid concentration according to the circadian rhythm on the performance, body composition, and nutrient balance of growing-finishing pigs
Source: PLoS One. 2021 Dec 23;16(12):e0261314. doi: 10.1371/journal.pone.0261314 (PMC8700050; doi:10.1371/journal.pone.0261314)
Supplement: S3 Table — (DOCX) [file pone.0261314.s003.docx]

**S3 Table. Body composition of the experimental pigs.**

| **Variables^1^** | **Mean** | **Minimum** | **Maximum** | **Standard deviation** | **Coefficient of variation (%)** |
| --- | --- | --- | --- | --- | --- |
| **Initial Conditions** |  |  |  |  |  |
| Body protein, kg | 3.189 | 1.994 | 4.274 | 0.516 | 16.210 |
| Body lipid, kg | 6.406 | 4.856 | 8.270 | 0.596 | 9.306 |
| Bone mineral content, kg | 0.529 | 0.410 | 0.643 | 0.048 | 9.148 |
| **Phase 1 (25–50 kg BW)** |  |  |  |  |  |
| Body protein, kg | 6.501 | 3.644 | 8.845 | 1.017 | 15.651 |
| Body lipid, kg | 10.554 | 7.235 | 14.267 | 1.626 | 15.412 |
| Bone mineral content, kg | 0.769 | 0.547 | 0.992 | 0.091 | 11.876 |
| Protein gain, g/day | 118.249 | 57.645 | 179.529 | 23.779 | 20.109 |
| Lipid gain, g/day | 148.050 | 68.403 | 276.860 | 48.686 | 32.884 |
| **Phase 2 (50–70 kg BW)** |  |  |  |  |  |
| Body protein, kg | 9.917 | 4.964 | 14.014 | 1.737 | 17.524 |
| Body lipid, kg | 15.904 | 8.371 | 23.420 | 3.493 | 21.968 |
| Bone mineral content, kg | 1.128 | 0.717 | 1.498 | 0.170 | 15.118 |
| Protein gain, g/day | 132.844 | 45.549 | 198.812 | 32.962 | 24.813 |
| Lipid gain, g/day | 208.276 | 43.727 | 352.045 | 77.495 | 37.208 |
| **Phase 3 (70–100 kg BW)** |  |  |  |  |  |
| Body protein, kg | 13.918 | 7.273 | 18.850 | 2.144 | 15.405 |
| Body lipid, kg | 25.254 | 11.147 | 35.803 | 5.528 | 21.889 |
| Bone mineral content, kg | 1.614 | 0.970 | 2.081 | 0.225 | 13.971 |
| Protein gain, g/day | 142.125 | 69.922 | 184.259 | 25.182 | 17.718 |
| Lipid gain, g/day | 331.899 | 99.116 | 531.985 | 87.575 | 26.386 |
| **Global body composition (25–100 kg BW)** |  |  |  |  |  |
| Protein gain, g/day | 131.054 | 62.605 | 183.321 | 22.709 | 17.328 |
| Lipid gain, g/day | 229.904 | 71.654 | 343.999 | 63.854 | 27.774 |

^1^ Body protein and lipid were estimated according to Pomar and Rivest (16) from lean and fat mass measured by dual-energy X-ray absorptiometry measurements (DXA).
