# Supplementary material for: Effects of sequential feeding with adjustments to dietary amino acid concentration according to the circadian rhythm on the performance, body composition, and nutrient balance of growing-finishing pigs
Source: PLoS One. 2021 Dec 23;16(12):e0261314. doi: 10.1371/journal.pone.0261314 (PMC8700050; doi:10.1371/journal.pone.0261314)
Supplement: S4 Table — (DOCX) [file pone.0261314.s004.docx]

**S4 Table. Nutrient balance of the experimental pigs.**

| **Variables^1^** | **Mean** | **Minimum** | **Maximum** | **Standard deviation** | **Coefficient of variation (%)** |
| --- | --- | --- | --- | --- | --- |
| **Phase 1 (25–50 kg BW)** |  |  |  |  |  |
| CP intake, g/day | 221.921 | 110.995 | 326.015 | 46.605 | 21.000 |
| N retention, g/day | 18.919 | 9.223 | 28.724 | 3.804 | 20.109 |
| N excretion, g/day | 16.587 | 6.209 | 27.014 | 4.579 | 27.609 |
| P intake, g/day | 6.819 | 3.468 | 9.961 | 1.422 | 20.858 |
| P retention, g/day | 2.814 | 1.297 | 4.588 | 0.656 | 23.332 |
| P excretion, g/day | 4.004 | 2.171 | 6.869 | 0.983 | 24.551 |
| N retention efficiency, % | 53.627 | 45.258 | 80.186 | 5.854 | 10.917 |
| P retention efficiency, % | 41.412 | 23.287 | 55.872 | 5.507 | 13.299 |
| **Phase 2 (50–70 kg BW)** |  |  |  |  |  |
| CP intake, g/day | 290.044 | 135.511 | 416.218 | 65.746 | 22.667 |
| N retention, g/day | 21.255 | 7.288 | 31.810 | 5.274 | 24.813 |
| N excretion, g/day | 25.152 | 12.994 | 40.853 | 6.421 | 25.532 |
| P intake, g/day | 9.386 | 4.444 | 13.372 | 2.103 | 22.413 |
| P retention, g/day | 3.968 | 1.501 | 6.278 | 1.059 | 26.698 |
| P excretion, g/day | 5.418 | 2.868 | 8.094 | 1.312 | 24.230 |
| N retention efficiency, % | 45.799 | 33.613 | 54.909 | 5.510 | 12.031 |
| P retention efficiency, % | 42.162 | 29.594 | 55.735 | 5.865 | 13.912 |
| **Phase 3 (70–100 kg BW)** |  |  |  |  |  |
| CP intake, g/day | 326.631 | 171.399 | 429.124 | 57.220 | 17.518 |
| N retention, g/day | 22.740 | 11.187 | 29.481 | 4.029 | 17.718 |
| N excretion, g/day | 29.561 | 10.685 | 42.123 | 7.072 | 23.923 |
| P intake, g/day | 11.059 | 5.834 | 14.418 | 1.927 | 17.424 |
| P retention, g/day | 4.715 | 2.428 | 6.801 | 0.851 | 18.057 |
| P excretion, g/day | 6.344 | 2.920 | 8.929 | 1.481 | 23.356 |
| N retention efficiency, % | 43.931 | 29.079 | 69.294 | 6.698 | 15.248 |
| P retention efficiency, % | 43.058 | 28.845 | 60.576 | 6.280 | 14.586 |
| **Global nutrient balance (25–100 kg BW)** |  |  |  |  |  |
| CP intake, g/day | 278.658 | 140.976 | 372.033 | 53.021 | 19.027 |
| N retention, g/day | 20.968 | 10.016 | 29.331 | 3.633 | 17.328 |
| N excretion, g/day | 23.723 | 12.145 | 34.436 | 5.544 | 23.369 |
| P intake, g/day | 9.081 | 4.633 | 12.099 | 1.706 | 18.791 |
| P retention, g/day | 3.826 | 1.748 | 5.202 | 0.681 | 17.821 |
| P excretion, g/day | 5.255 | 2.776 | 7.610 | 1.143 | 21.765 |
| N retention efficiency, % | 47.221 | 40.357 | 56.212 | 4.110 | 8.704 |
| P retention efficiency, % | 42.346 | 34.689 | 50.697 | 3.595 | 8.491 |

^1^ CP, crude protein; N, nitrogen; P, phosphorus. Body protein was estimated according to Pomar and Rivest (16) from lean mass measured by dual energy X-ray absorptiometry measurements (DXA) and converted to nitrogen. The total body phosphorus value was estimated according to Letourneau-Montminy, Narcy (17). Nitrogen and phosphorus efficiency were obtained by the relationship between deposition and intake of phosphorus and protein, respectively.
